# Epstein-Barr Virus (EBV)-Associated Haemophagocytic Syndrome

**DOI:** 10.4084/MJHID.2012.008

**Published:** 2012-01-25

**Authors:** Lorenza Torti, Luigi M. Larocca, Giuseppina Massini, Annarosa Cuccaro, Elena Maiolo, Rosaria Santangelo, Maria Bianchi, Mariano Alberto Pennisi, Stefan Hohaus, Luciana Teofili

**Affiliations:** 1 Department of Hematology, Catholic University, Rome, Italy; 2 Department of Pathology, Catholic University, Rome, Italy; 3 Department of Microbiology, Catholic University, Rome, Italy; 4 Department of Anaesthesiology, Catholic University, Rome, Italy

## Abstract

We describe the case of a 17- year old female who developed fatal haemophagocytic syndrome (HPS) one month following acute infection caused by Epstein-Barr virus (EBV). Despite initiation of treatment and reduction of EBV load, laboratory signs of HPS as severe cytopenia, hypofibrinogenemia, hyperferritinemia and hypertriglyceridemia persisted, and the patient died of multiorgan failure. HPS is a rare, but life-threatening complication of EBV infection.

## Introduction

The haemophagocytic syndrome (HPS) represents a severe and aggressive hyperinflammatory disease with major diagnostic and therapeutic difficulties.^1^–^3^ HPS is a spectrum of inherited and acquired conditions with disturbed immune regulation which results into a dysregulated activation and proliferation of lymphocytes. Haemophagocytosis is part of a sepsis-like syndrome caused by severe hypercytokinemia as result of a highly stimulated but ineffective immune response. Cardinal symptoms are prolonged fever, cytopenia affecting at least two of three lineages, hepatosplenomegaly and coagulopathy with hypofibrinogenemia. Diagnostic criteria for this disorder are shown in Table 1 edited by the Histiocyte Society.^2^

HPS may be the consequence of a familial immune dysregulatory disorder or be associated with a number of different infections, autoimmune disorders or malignancies, like lymphomas or carcinomas.^4^,^5^ Frequent triggers are infectious agents, especially virus of the herpes group.^6^ Among the infectious agents associated with HPS there are EBV, Cytomegalovirus, Parvovirus, Varicella zoster, Herpes-simplex as well as Herpes-Virus 8 and HIV infection alone or in combination. EBV is the most common infectious agent.

EBV-associated haemophagocytic syndrome has a relatively high incidence in Asian countries and particularly in Japan.^3^ Here we present a case of EBV-associated haemophagocytic syndrome in a young Italian women that was fatal despite prompt medical treatment.

## Case Presentation

A 17 year-old white woman was admitted to our hospital for fever, fatigue and abdominal discomfort. Her medical history showed no relevant data; in particular no recurrent infectious episodes were documented except for the common childhood diseases. One month before the admission, the patient suffered from infectious mononucleosis. She had developed a widespread skin rash during antibiotic therapy with amoxicillin given for fever. A that time, the monospot test resulted positive and the patient received corticosteroids with symptoms improvement.

On admission, the physical examination showed splenomegaly without palpable lymphadenopathy. Laboratory evaluation revealed a severe pancytopenia: platelet count was 17× 10^6^/mmc, hemoglobin was 7,2 g/dl and she had a leucocyte count of 230/mmc. Lactate dehydrogenase was strongly increased to 1570 UI/l, while GPT was 217 U/l and bilirubin was elevated to 4,6mg/dl (Table 2). Clotting tests showed severe hypofibrinogenemia with a normal value of international normalized ratio (INR) and of activated partial thromboplastin time (aPTT). A bone marrow aspiration for the suspect of an acute hematological proliferative disease was performed, but was not diagnostic. Ferritin levels were high (6421 ng/ml). Serological studies for HIV 1,2, hepatitis B and C, cytomegalovirus, parvovirus were negative. Both IgG and IgM anti- viral capsid antigen (VCA) EBV antibodies were positive, while anti-EBV nuclear antigen were negative, indicating a recent primary EBV infection. Amplification of EBV-DNA from whole blood by polymerase chain reaction (PCR) yielded 930.000 copies/ml. As the patient presented signs of an acute colecystitis, she was treated with trans-hepatic drainage of the gall-bladder.

Acute acalcolous colecystitis (AAC) observed in our patient, reflected the severe inflammatory response associated with the primary EBV infection. In addition, the bile stasis can also been implicated in the gallbladder inflammation pathogenesis. Moreover, also EBV-induced hepatitis has been recognized as an important cause of cholestasis. Finally, another possible pathogenetic mechanism, may be the direct invasion of the gallbladder from the EBV-virus. AAC may develop during the course of acute EBV infection, especially in patients with cholestatic hepatitis.^6^,^7^

**Figure 1:**
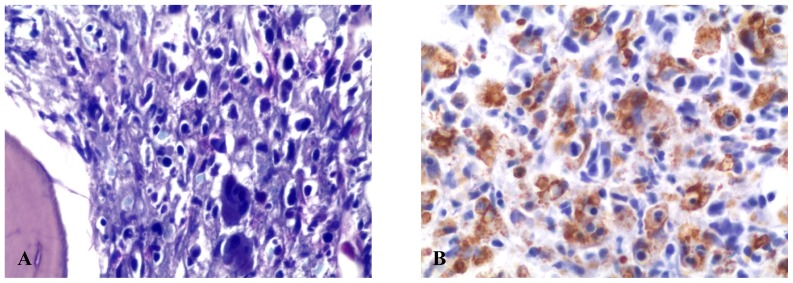
A) Giemsa staining of the bone marrow biopsy. Morphological signs of hemophagocytosis with numerous macrophages engulfing various cellular elements in their cytoplasm. B) Immunohistochemical analysis for CD68 in the bone marrow biopsy utilizing the PGM-1 antibody.

Clinical conditions of our patient rapidly deteriorated and she was transferred to the intensive care unit on day 2 following admission. Hemophagocytic syndrome was suspected and confirmed by a second bone marrow examination (Table 3). Bone marrow cytology and histology revealed several histiocytes with phagocytosis of erythrocytes and platelets (Figure 1). The bone marrow biopsy showed phagocytic cells with engulfed haemopoietic elements. The presence of large sized T lymphocytes in the biopsy raised the suspicion of EBV-related T-lymphoproliferative disease.

**Figure 2:**
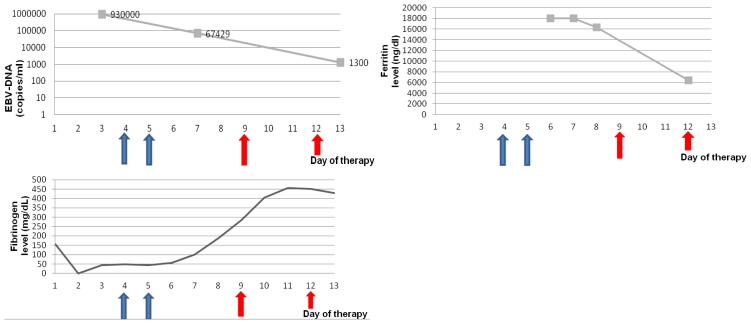
Changes in laboratory parameters during the clinical course of the patient. The blue arrays indicate the administration of immunoglobulins (1 g/kg) on days 4 and 5, the red arrows indicate therapy with etoposide (150 mg/m^2^).

The patient was immediately started on high doses of corticosteroids and immunoglobulins (1g/kg for two days). As the clinical conditions did not improve, treatment with etoposide was started according to the HLH-94 protocol. Etoposide was administered in a dose of 150 mg/m 2, on day 9 and day 12. EBV copy numbers slowly decreased, and laboratory signs of HPS as severe hypofibrinogenemia and hyperferrtinemia improved (Figure 2), but pancytopenia persisted and the patient was supported with massive transfusions of blood and fresh frozen plasma, granulocyte-colony stimulating factor (G-CSF), and antibiotics. The patient died in multiorgan failure on day 15 following admission.

## Discussion

Haemophagocytic syndrome (HPS) is the most severe complication of infectious mononucleosis. HPS is a highly fatal disease if untreated or treated late. Diagnosing HPS is a challenge, as there is no single simple diagnostic test, and it remains a syndromic disorder with a combination of typical findings being often initially absent but developing with progressive disease. Because of diagnostic difficulties, diagnosis of HPS is often delayed and only established when the patient is already critically ill and transferred to the intensive care unit, as in our case.

As EBV responds poorly to antiviral agents, treatment is aimed at controlling the lymphocyte/macrophage activation and proliferation. In addition to corticosteroids and immunoglobulins, treatment of EBV-associated HPS often requires administration of etoposide.^8^,^9^ Repetitive administration of a myelosuppressive agent in an already severe cytopenic patient with a high risk for additional infectious complications is not trivial, and should be limited to centers experienced in treatment of acute haematological diseases.

Since HPS is rare, no data are available from randomized controlled clinical trials testing potential different treatment approaches. As T cell dysregulation appears to be involved in the pathogenesis of both primary and secondary HPS, treatment with anti-thymocyte globulins (ATG) has been reported as an alternative to etoposide-based therapy in a single center study on patients with familial HPS.^10^ An on-going trial of the Histiocyte Society studies the early addition of cyclosporine to the etoposide-based regimen.^11^ An additional therapy for patients with progressive EBV-associated HPS may be rituximab, at it can eliminate EBV-infected B cells.^12^

Bone marrow histology raised the suspicion of a T cell lymphoma in our patient. Lymphomas of T-cell and NK-cell origin are among the most common neoplasias associated with secondary malignancy-associated HPS.^4^,^5^,^11^ This association has been more frequently been reported in Asian countries where the incidence of EBV-associated lymphomas appear to be significantly higher. In fact some studies describe fulminant EBV-T cell lymphoproliferative disorders following acute EBV-infection.^13^ However, the suspicion in our patient was not substantiated by other signs of a lymphoproliferative disease, although we cannot rule out that the EBV-induced lymphoproliferation in our patient was already indication of transformation into a malignant disease. In this case, only the assessment of the monoclonal nature of the EBV-infected T cell expansion could have definitely indicate the transformation in lymphoma.

In conclusion, our case highlights that hemophagocytic syndrome can complicate acute EBV-infection, and should raise the awareness for this rare life-threatening syndrome in order to promptly start appropriate treatment.

## Figures and Tables

**Table 1:** Diagnostic criteria for HPS: One of two criteria should be present for correct diagnosis^4^

Molecular diagnosis of familial haemophagocytosis (Pathologic mutations of Perforin (PRF1), SH2D1A/SAP, UNC13D, Syntaxin 11 (STX11), MUNC18-2, Ras-related Protein Rab27a (RAB27a)) **Or** Five out of eight diagnostic criteria listed below are fulfilled: Fever ≥38.5°C Splenomegaly Cytopenias (affecting at least 2 of 3 lineages in the peripheral blood): Hemoglobin <9 g/dl (in infants <4 weeks: hemoglobin <10 g/dl) Platelets <100×103/ml Neutrophils <1×103/ml Hypertriglyceridemia (fasting, > 265 mg/dl) and/or hypofibrinogenemia (<150 mg/dl) Hemophagocytosis in bone marrow or spleen or lymph nodes or liver Low or absent NK-cell activity Ferritin > 500 ng/ml Elevated Soluble CD25 (alpha chain of soluble IL-2 receptor)

**Table 2:** Laboratory data of the patient

Complete blood cell counts		Normal range
GB	0.23 × 10^9^/l	4–10 × 10^9^/l
Platelets	**17 × 10^9^/l**	150–450 × 10^9^/l
Hemoglobin	**7,2 g/dl**	12–14 g/dl

**Biochemistry**		

LDH	**1570 UI/l**	230–460 UI/l
GPT	**217 UI/l**	7–45 UI/l
Creatinina	0,9 mg/dl	7–1,2 mg/dl
BUN	18 mg/dl	10–23 mg/dl
Bilirubin	**4.6 mg/dl**	0.3–1.2 mg/dl
Ferritin	**6421 ng/ml**	11–307 ng/ml

**Coagulation tests**		

PT	10,2 sec.	
Fibrinogen	**43 mg/dl**	200–400 mg/dl
INR	0,97	0,8–1,2

**Immunological tests**		

IgG	903 mg/dl	700–1600 mg/dl
IgM	96 mg/dl	40–230 mg/dl
IgD	119 U/ml	<110 U/ml
IgE	37 UI/ml	<100 UI/ml
IgA	154 mg/dl	70–400 mg/dl

**Table 3:** Bone marrow cytology.

**Myelogram**	
- Erytroid lineage	4%
- Granulocytic lineage	35%
- Lymphocytes	54%
- Eosinophils	2%
- Monocytes	5%

Comment. Several histiocytes with signs of phagocytosis of erythrocytes and platelets

## References

[1] Fisman DN (2000). Hemophagocytic syndromes and infection. Emerg Infect Dis.

[2] Henter JI, Horne A, Aricó M, Egeler RM, Filipovich AH, Imashuku S, Ladisch S, McClain K, Webb D, Winiarski J, Janka G (2007). HLH-2004: Diagnostic and therapeutic guidelines for hemophagocytic lymphohistiocytosis. Pediatr Blood Cancer.

[3] Janka GE (2007). Hemophagocytic syndromes. Blood Rev.

[4] Tong H, Ren Y, Liu H, Xiao F, Mai W, Meng H, Qian W, Huang J, Mao L, Tong Y, Wang L, Qian J, Jin J (2008). Clinical characteristics of T-cell lymphoma associated with hemophagocytic syndrome: comparison of T-cell lymphoma with and without hemophagocytic syndrome. Leuk Lymphoma.

[5] Gallipoli P, Drummond M, Leach M (2009). Hemophagocytosis and relapsed peripheral T-cell lymphoma. Eur J Haematol.

[6] Shaukat A, Tsai HT, Rutherford R, Anania FA (2005). Epstein–Barr virus induced hepatitis: an important cause of cholestasis. Hepatol Res.

[7] Attilakos A, Lagona E, Sharifi F, Voutsioti A, Mavri A, Markouri M (2007). Epstein-Barr Virus Infectious Mononucleosis Associated with Acute Acalculous Cholecystitis. Infection.

[8] Rouphael NG, Talati NJ, Vaughan C, Cunningham K, Moreira R, Gould C (2007). Infections associated with haemophagocytic syndrome. Lancet Infect Dis.

[9] Imashuku S, Kuriyama K, Teramura T, Ishii E, Kinugawa N, Kato M, Sako M, Hibi S (2001). Requirement for etoposide in the treatment of Epstein-Barr virus-associated hemophagocytic lymphohistiocytosis. J Clin Oncol..

[10] Mahlaoui N, Ouachee-Chardin M, de Saint BG (2007). Immunotherapy of familial hemophagocytic lymphohistiocytosis with antithymocyte globulins: a single-center retrospective report of 38 patients. Pediatrics.

[11] Jordan MB, Allen CE, Weitzman S, Filipovich AH, McClain KL (2011). How I treat hemophagocytic lymphohistocytosis. Blood.

[12] Milone MC, Tsai DE, Hodinka RL (2005). Treatment of primary Epstein-Barr virus infection in patients with X-linked lymphoproliferative disease using B-cell-directed therapy. Blood.

[13] Awaya N, Adachi A, Mori T, Kamata H, Nakahara J, Yokoyama K, Yamada T, Kizaki M, Sakamoto M, Ikeda Y, Okamoto S (2006). Fulminant Epstein-Barr virus (EBV)-associated T-cell lymphoproliferative disorder with hemophagocytosis following autologous peripheral blood stem cell transplantation for relapsed angioimmunoblastic T-cell lymphoma. Leuk Res.

